# Biomedically Relevant Applications of Bolaamphiphiles and Bolaamphiphile-Containing Materials

**DOI:** 10.3389/fchem.2020.604151

**Published:** 2021-01-20

**Authors:** Jake R. Hughes, Alyssa S. Miller, Camryn E. Wallace, Gopi Nath Vemuri, Peter M. Iovine

**Affiliations:** Department of Chemistry and Biochemistry, University of San Diego, San Diego, CA, United States

**Keywords:** bolaamphiphiles, biomaterials, bolaform amphiphile, gene delivery, antimicrobials, prodrugs

## Abstract

Bolaamphiphiles (BAs) are structurally segmented molecules with rich assembly characteristics and diverse physical properties. Interest in BAs as standalone active agents or as constituents of more complex therapeutic formulations has increased substantially in recent years. The preorganized amphiphilicity of BAs allows for a range of biological activities including applications that rely on multivalency. This review summarizes BA-related research in biomedically relevant areas. In particular, we review BA-related literature in four areas: gene delivery, antimicrobial materials, hydrogels, and prodrugs. We also discuss several distinguishing characteristics of BAs that impact their utility as biomedically relevant compounds.

## Introduction

Synthetic bolaamphiphiles (BAs) are segmented amphiphilic molecules that take structural inspiration from archaeal membrane lipids (Fuhrman et al., 1992; Benvegnu et al., 2008). Traditionally, BAs are comprised of two polar head groups linked through a hydrophobic tether. The polar head groups can be uniform or varied (i.e., asymmetric BAs) but the triblock segmented structure is required. An increasing number of “inverted bolaamphiphiles” have appeared in the literature featuring a polar central spacer flanked by nonpolar head groups (Hajj et al., 2020; Wei et al., 2020; Xiong et al., 2020). Given the possible confusion in regard to the terminology of these types of molecules, it seems important to define a BA based on its segmented amphiphilic structure (A-B-A), not on the ordering of the hydrophilic and hydrophobic segments. The distinction between BAs and triblock copolymers is one of molecular weight. Historically this line was clear–BAs were low molecular weight while triblock copolymers were high molecular weight (polymeric). More recently, however, the line is blurring with mixed systems featuring dendritic hydrophilic capping groups linked through a low molecular weight hydrophobic tether (Rashmi et al., 2018).

Fuhrhop was instrumental in advancing the field of BAs especially in regard to their rich self-assembly behavior. In 1984, Fuhrhop and Fritsch reported a hydrophobically linked paraquat and used the term bolaamphiphile in the body of the paper (Fuhrhop and Fritsch, 1984). In 1986, the same authors, for the first time, used the term “bolaamphiphile” in a journal article title (Fuhrhop and Fritsch, 1986).

Several reviews have summarized traditional areas of BA research. In a highly cited 2004 review, Fuhrhop and Wang (2004) cover synthetic methods, self-assembly characteristics, as well as applications in areas such as pore-forming and electron-conducting materials. Meister and Blume (2007) review focused exclusively on self-assembly as did a later review by Dhasaiyan and Prasad (2017). In 2013, Nuraje et al. shifted their attention toward BA applications (Nuraje et al., 2013).

Current research in BAs is heavily weighted toward delivery applications (Fariya et al., 2014) as well as exploiting BA membrane-active properties. One approach to categorizing the large amount of literature is to focus on what role the BA plays. Is the BA a standalone agent or is it a component of a larger delivery matrix? A good example of a BA acting as a standalone biologically-active agent is pore-forming BAs. Seminal work by Gokel and Murillo (1996) and a recent review (Gill et al., 2020) summarizes progress in using pore formation as an antimicrobial strategy. Others have pursued membrane active properties with a wide variety of BA and BA-like structures (Fyles et al., 2001; Cameron et al., 2002; O'Neil et al., 2007). Research involving BAs as an active component of a more complex formulation is expanding. This is especially true in gene delivery where multicomponent lipid nanoformulations are common.

**Figure 1:**
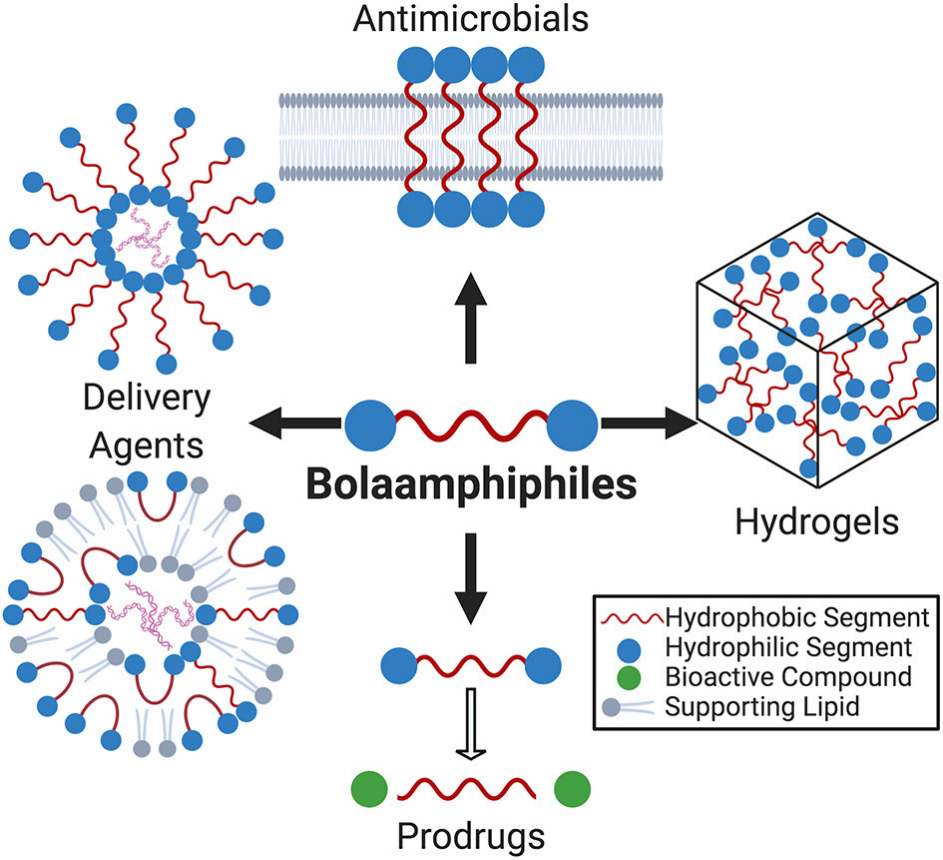
Due to their segmented amphiphilicity and rich self-assembly behavior, bolaamphiphiles (BAs) have emerged as versatile building blocks for therapeutically relevant biomaterials. This review focuses the impact of BAs in drug delivery, prodrugs, hydrogels, and antimicrobials.

The goal of this review is to summarize developments in biomedically relevant BA research. Figure 1 provides an overview of the research topics covered. We have attempted to avoid significant overlap with other relevant reviews (Fariya et al., 2014) but, in some cases, duplications were unavoidable.

## Bolaamphiphile Specific Considerations Relevant to Biomedical Applications

### Segmented Amphiphilicty

The most distinguishing feature of a BA is its segmented amphiphilic structure akin to a low molecular weight triblock copolymer. As in multiblock copolymers, self-organization of BAs is likely driven by the minimization of contacts between the disparate structural domains and the gain in noncovalent interactions. The BA's intrinsically ordered structure, then, leads to its complex self-assembly behavior. Yan et al. has studied packing parameters for a series of symmetric BAs as a function of pH and additives. They found that that the morphology of the aggregate can be predicted using methods developed for conventional surfactants (Yan et al., 2007).

The impact of segmented amphiphilicity extends beyond nanostructures. For example, incorporating dendritic hydrophilic head groups (as compared to a single hydrophilic moiety) creates opportunities to combine self-assembly with multivalent ligand presentation. Here, the segmented amphiphilicty facilitates particle organization while the multivalent hydrophilic segments enhance biological function.

An important outcome of BA segmented amphiphilicity is the ability to form monolayer membranes (MLM). *Archaea* microorganisms famously utilize ether-linked BA lipids to assemble and stabilize a range of membrane organizations but especially the monolayer type (Benvegnu et al., 2008).

**Figure 2:**
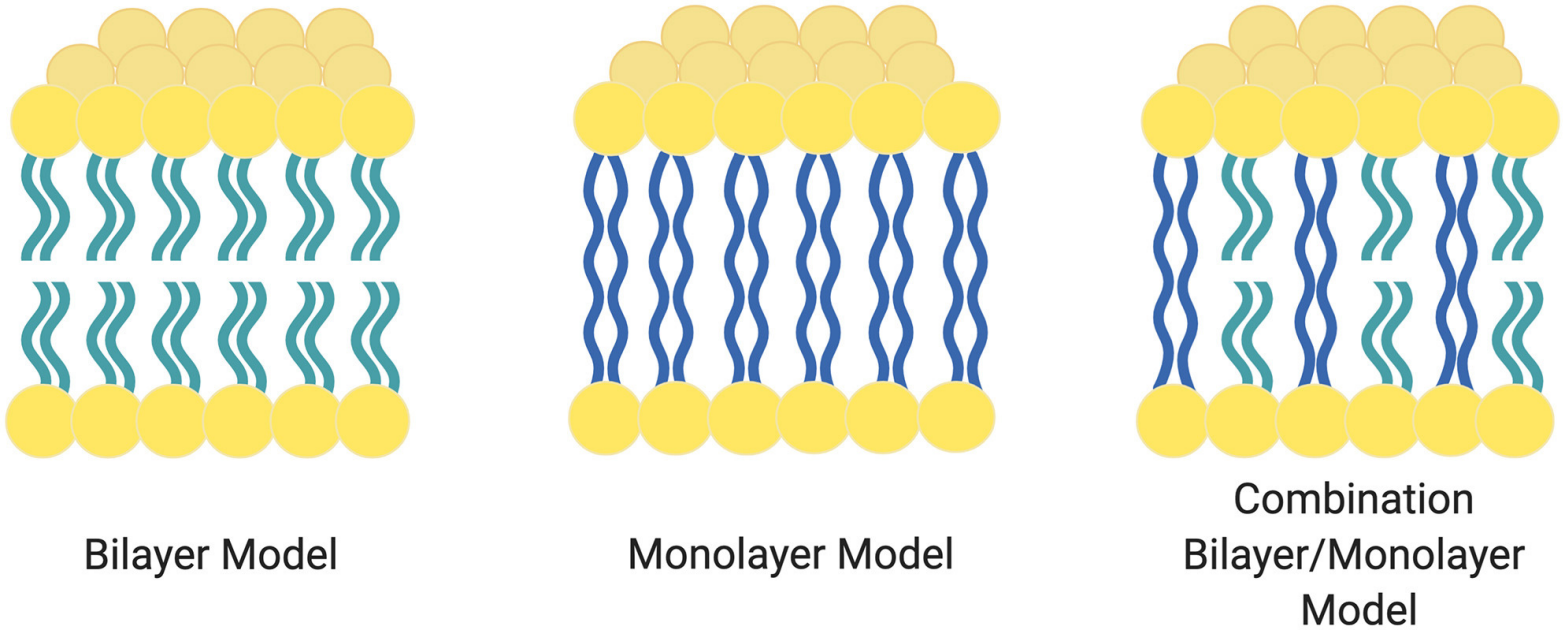
Various forms and combinations of Archaea domain membranes. The left most image shows a traditional bilayer with no bridging chains. The center image shows a monolayer membrane. The right most image represents a combination of both bridging and traditional lipids. Figure adapted from Benvegnu et al. (2008).

As shown in Figure 2, the balance of the central hydrophobic domain and the neutral hydrophilic domains leads to stabilized MLM or mixed membrane architectures. The ether linkages connecting the segments (as compared to esters) stabilize the membranes under extreme environmental conditions.

In some situations membrane stabilization is desirable (e.g., drug delivery with liposomes) but in other applications destabilization becomes the goal. The use of BAs as ion-conducting species was one of the earliest and most robust lines of research inside the synthetic amphiphile community (Fuhrhop and Wang, 2004). Here, pore formation and ion conductance are related to the membrane insertion propensity of the amphiphile. There has been a recent shift, however, in utilizing the pore forming capabilities of BAs not for ion transport but rather for antimicrobial applications. Here, as summarized by Gill et al. (2020), the BA insertion has a dual mode of action: increase membrane instability and disrupt ion homeostasis. Fundamentally, the biological properties of these BA-based antimicrobials rely on the segmented amphiphilicity that facilitates efficient membrane insertion.

Traditional symmetric BAs contain three segments with the two flanking segments being identical. Asymmetric BAs, on the other hand, have three unique segments with potentially different degrees of amphiphilicity, charge, and/or size. From a self-assembly point of view (but not from a synthetic point of view), asymmetric BAs are attractive as they can encode a higher density of information vs. symmetric BAs. For example, Jain et al. (2010, 2012) studied a series of asymmetric BAs that featured both a neutral sugar hydrophilic segment and a nucleic acid binding cationic segment. In the presence of nucleic acid, the BAs self-assemble into defined particles with the larger sugar segments oriented toward the bulk water phase. Importantly, the asymmetry of the BA allowed for pre-programming of the monolayer organization.

**Figure 3:**
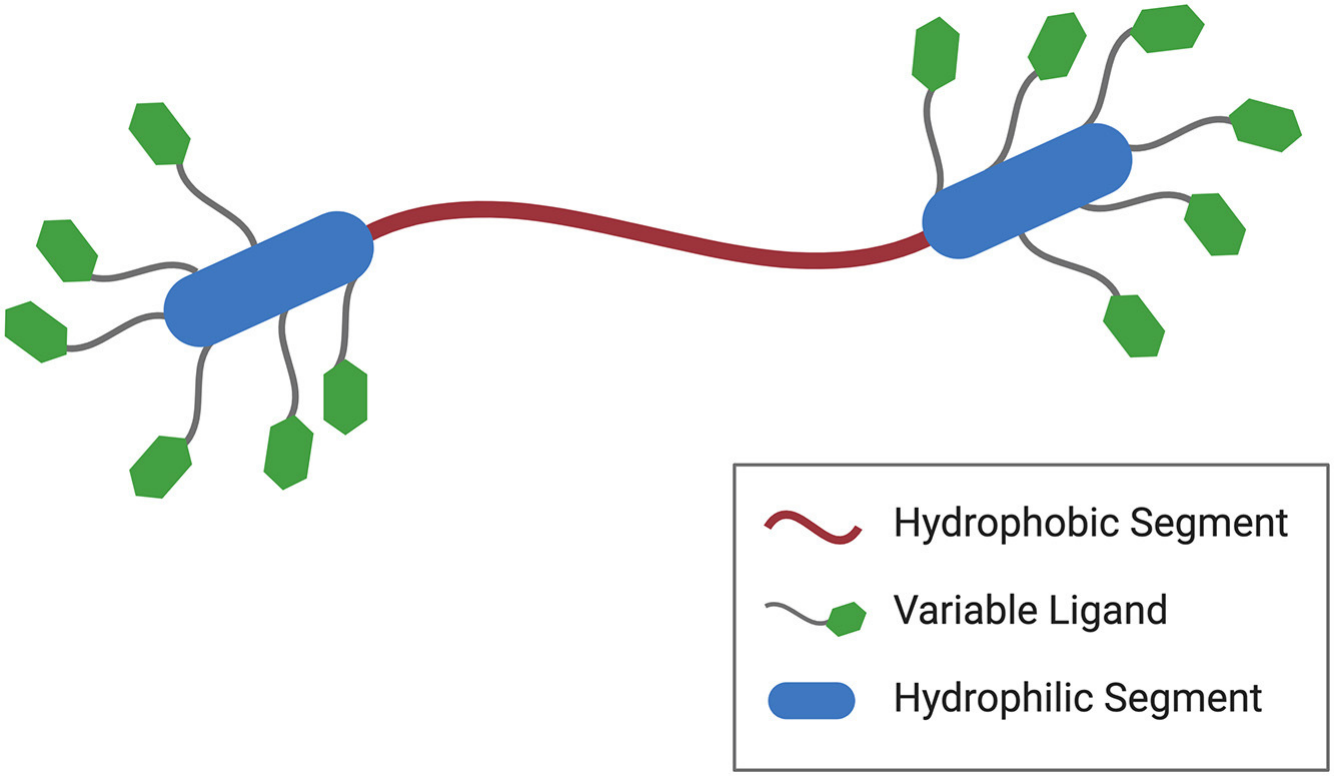
Generalized BA structure featuring not only segmented amphiphilicity but also multivalent domains. In this particular case, the hydrophilic segments carry multiple copies of a functional ligand or stimuli-responsive group potentially leading to cooperative binding interactions.

Another notable feature of segmented amphiphilicity is the organization of functional groups into dense clusters. Figure 3 illustrates this point. It shows a prototypical symmetric BA with flanking hydrophilic segments. Each hydrophilic segment carries multiple copies of a functional group or ligand that can bind, possibly in a cooperative manner, to a receptor or target. In this way, multivalent effects used to achieve higher efficacy in the selected application. The delivery of genes or gene therapeutics via nonviral constructs, for example, typically relies on the electrostatic complexation of a cationic carrier with the anionic payload. Increasing evidence suggests that cooperativity and polyelectrolyte effects are important factors in determining the efficiency for certain key steps of the delivery process (Drean et al., 2017; Kumar et al., 2020). BAs, then, seem well suited for these applications given their innate segmented structures that facilitate dense functional group placement.

### Stability of BA-Containing Nanostructures

**Figure 4:**
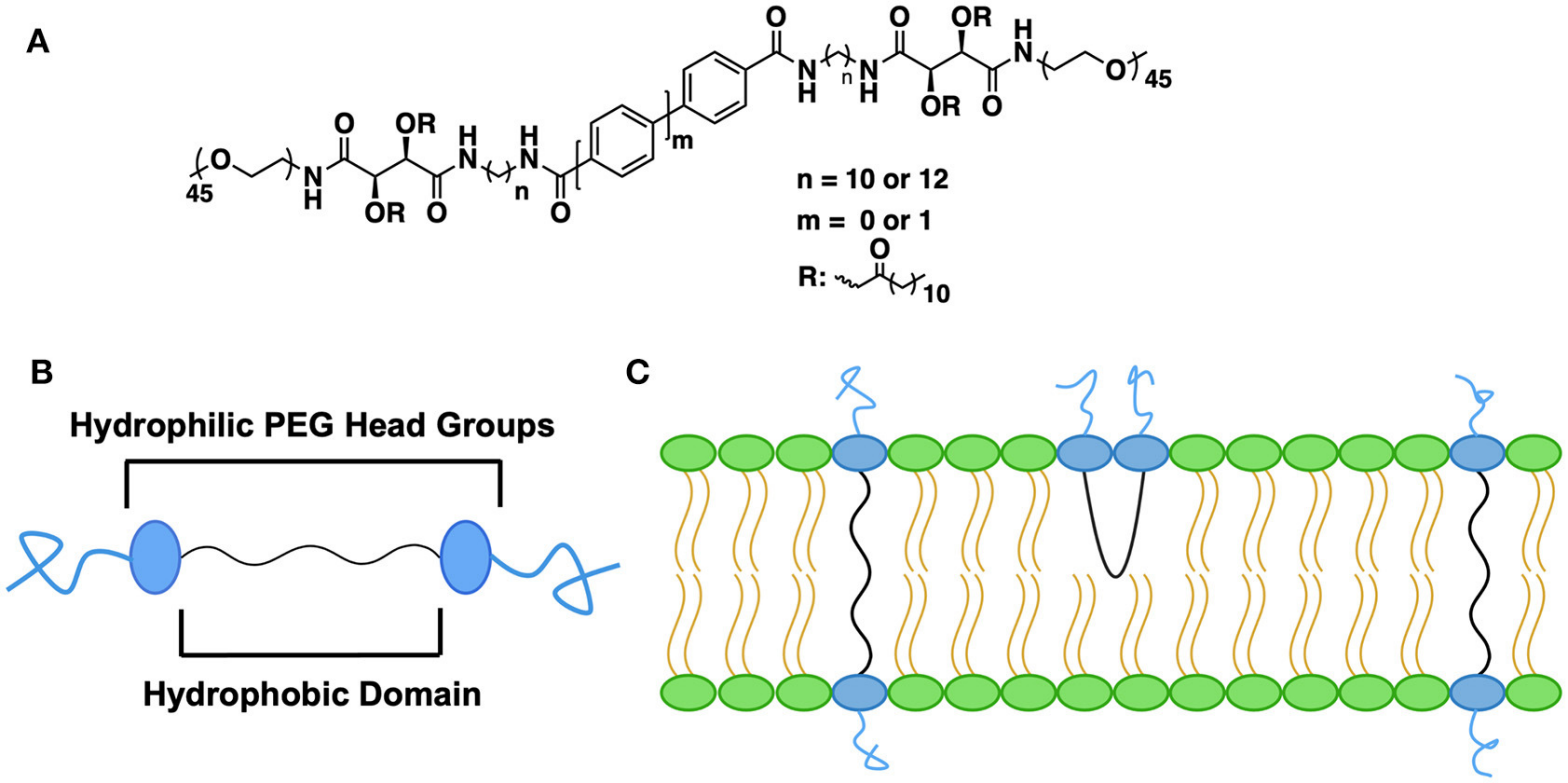
Impact of PEG-containing BAs on liposomal stability. **(A)** Chemical structures of amphiphilic PEG-BAs, **(B)** Structural cartoon highlighting the segmented as well as the terminal PEG groups, **(C)** Two different PEG-BA conformations in the lipid layer. The membrane-spanning orientation is preferred over U-shaped due to the presence of rigid aryl groups in the hydrophobic segment. Figure adapted from Zhang et al. (2016).

Another cross-cutting theme that emerges from the literature is the ability of BAs to impact stability of nanostructures either alone or as a component in a more complex formulation. Archaea-like or archaea-inspired BAs are well-known in this regard. Their membrane spanning ether linked lipid backbone along with hydrophilic head groups stabilize the liposomal membrane (Benvegnu et al., 2005; Mahmoud et al., 2015; Jacobsen et al., 2017; Koyanagi et al., 2017; Leriche et al., 2017, 2018). Liposomal stability enhancement is not limited to archaea-type BAs. Elegant work by the Uhrich group compared the integrity of the liposomal PEG stabilizing layer using both PEGylated BAs and conventional PEGylated lipids (Zhang et al., 2016). The authors found similar liposomal stability for both conventional and BA-based PEGylated lipid but enhanced retention of the BA upon dilution (see Figure 4). Given the large design landscape for both the hydrophobic and hydrophilic segments of the BA, novel approaches to stabilization of liposomal nanostructures remains a fertile area.

**Figure 5:**
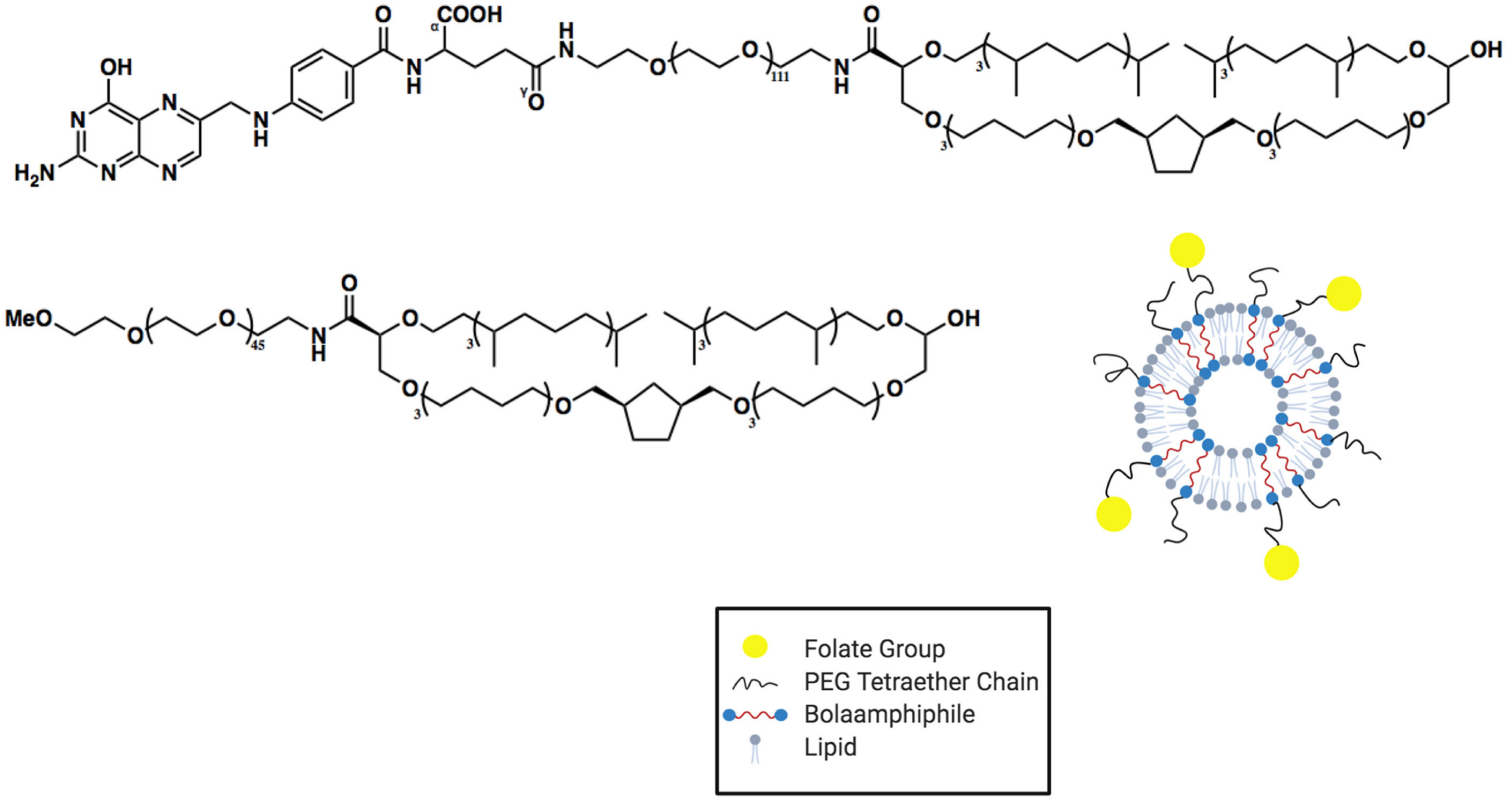
Structure and cartoon versions of archaeosomes synthesized by Jiblaoui et al. (2016). Two distinct membrane-spanning BAs were used in the assembly of these archaeosomes: a PEG functionalized BA as well as a folate-carrying BA.

The Benvegnu group has also worked extensively in the area of synthetic BA archaeasomes (Benvegnu et al., 2005; Jiblaoui et al., 2016; Resnier et al., 2019). In 2016 (Jiblaoui et al., 2016), they reported the synthesis and characterization of folate-conjugated archaeosomes (see Figure 5). These materials were developed to not only enhance the stability of the liposomal formulation but also take advantage of cell ligand-receptor targeting. In order to accomplish both of these objectives, tetraether-linked lipids were synthesized and conjugated, through a short PEG chain, to folate. The synthesis of the folate-conjugated tetraether lipid was accomplished in four high yielding steps (not including the ether-linked lipid synthesis).

The impact of sugar-capped BAs on bilayer stability has also been studied (Gatard et al., 2013; Nasir et al., 2016). These fascinating biosurfactants can be synthesized in just two steps: a glycosylation followed by the metathesis of a terminal alkene. The metathesis reaction extends the hydrophobic central linker and symmetrizes the BA. The authors found that the type of sugar used as the end-capping unit has little impact on the membrane active properties. However, the presence of sterols in the membrane (i.e., cholesterol) in the membrane attenuates the insertion of the BA. Mechanistically, it is thought that the cholesterol promotes tighter packing of the bilayer thereby restricting the BA insertion.

### Toxicity Profile of BA-Containing Biomaterials

Early work in BAs focused on membrane insertion and pore formation–events that potentially disrupt or destabilize membranes. Balancing these attractive membrane active properties with potential off target toxicity concerns is a critical interplay especially in application areas like gene delivery and antimicrobials. Interestingly, though, many studies reviewed herein find rather low cytotoxicity of their biomedically relevant BAs. For example, in a study focusing on siRNA delivery using BAs, the Guan group purposely chose a hydrophobic linker that was shorter than the native bilayer thereby disfavoring insertion (Zeng et al., 2015). In addition, a dendritic headgroup was used to disfavor membrane interactions via a U-shape conformation. Both of these design criteria, length of the hydrophobic spacer and the nature of the hydrophilic head group, are key factors in mitigating toxicity. In another study focused on the delivery of nanocomplexes containing transcription factor decoys (TFD) and cationic BAs to bacteria, it was found that cardiolipin plays a key role in facilitating delivery to the bacterial membrane. Cardiolipin is highly anionic lipid found in bacterial membranes but, importantly, not in eukaryotic plasma membranes. MTT and hemolysis assays using the BA-based nanocomplexes show satisfactory safety profiles.

A large number authors cite favorable BA safety profile as a justification for their use in biomedical applications. Given the diversity of BA structures found in the literature, as well as their complex self-assembly processes that can alter their mode of action, safety should be evaluated and not assumed. Importantly, though, as pointed out above, rational design can be used to mitigate potential safety issues thereby increasing the potential of BAs in biomedically relevant areas.

## Bolaamphiphiles in Gene Delivery

**Figure 6:**
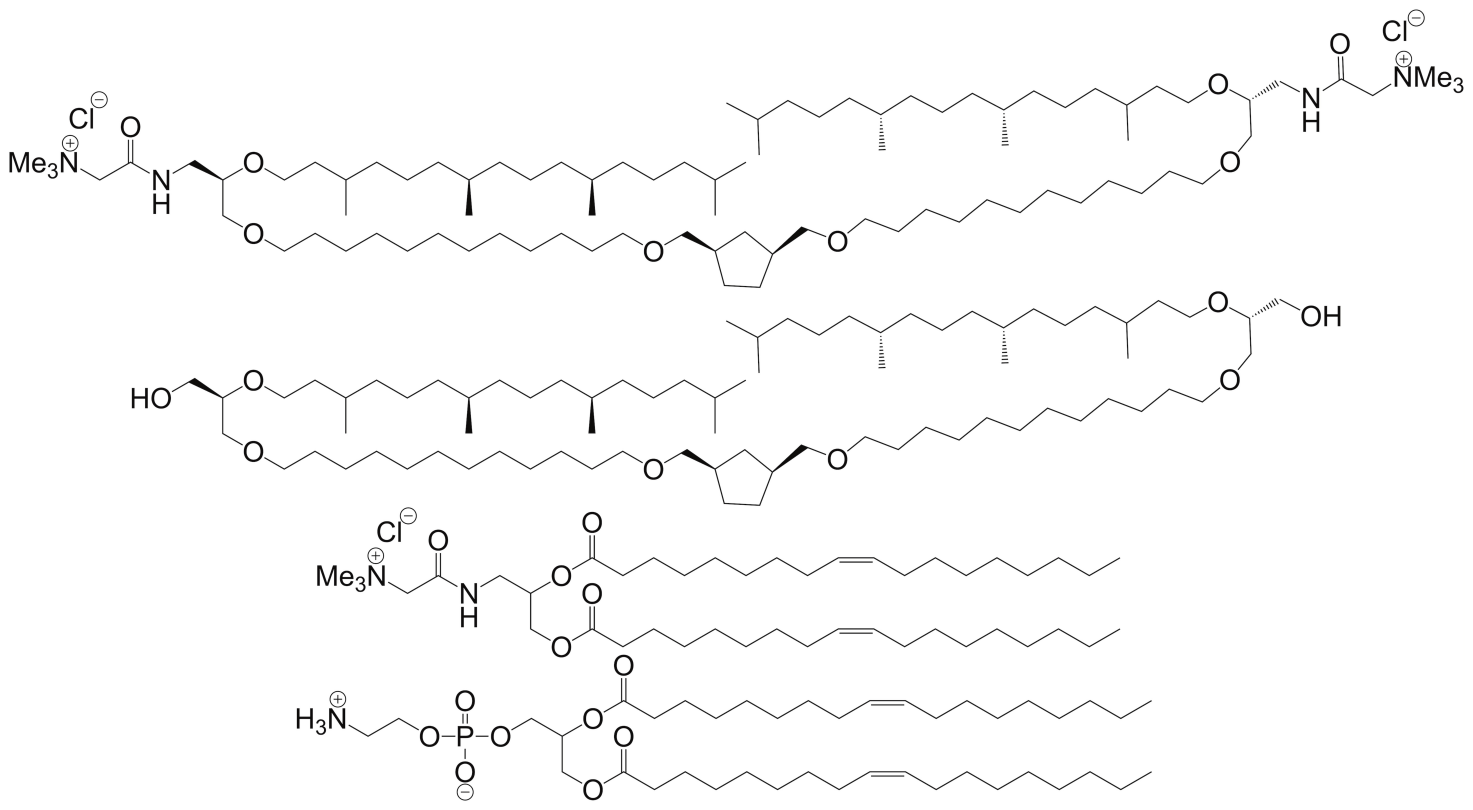
Archaeosome forming BAs (top two structures) are co-formulated with helper lipids (bottom two structures) in order to form monolayer-type micelles. Reproduced from Rethore et al. (2007), Royal Society of Chemistry.

Synthetic BAs take inspiration from archaeobacterial-type lipids whose ether-linked structure promotes monolayer organization rather than a traditional bilayer (Benvegnu et al., 2008). Some of the earliest work utilizing BAs in gene delivery involved archaeal-like lipids. In 2007, the Benvegnu group reported a new class of cationic ether-linked BA that, when co-formulated with more traditional helper lipids, forms structurally distinct archaeosomes (see Figure 6) (Rethore et al., 2007).

The authors demonstrate how cationic BA-type lipids can be used in concert with traditional bilayer-forming lipids to adjust the properties of the final lipid-DNA complex. It is important to note that prior to Benvegnu's work in archaeal-like cationic lipids, several papers describe the use of BA-like structures for *in vitro* gene delivery. For example, in 2001 and again in 2003, Vierling et al. describe *in vitro* gene delivery using galactosylated spermine and polyamine constructs, respectively (Gaucheron et al., 2001; Fabio et al., 2003). Denoyelle et al. (2006) also describe a series of dissymmetric hemifluorocarbon BAs that efficiently condense DNA and exhibit low cytotoxicity with COS-7 cells while providing high transfection efficiency. In both cases, the authors categorize their compounds of interest as BAs, however, these compounds feature a branched hydrophobic domain rather than a linear architecture observed in traditional BAs.

**Figure 7:**
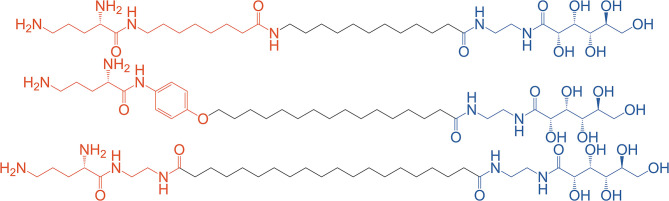
Asymmetric BA with varying chain lengths containing a uniform gluconic acid (blue) and varying ornithine containing moieties (red). Reproduced from Jain et al. (2010), American Society of Chemistry.

Klymchenko et al. have pioneered the synthesis of asymmetric BAs for DNA complexation either as a single agent (bolaplexes) or co-formulated with helper lipids. In 2010, they reported the synthesis and complexation of DNA by asymmetrical BAs featuring ornithine and gluconic acid polar head groups (Jain et al., 2010) (see Figure 7) whereas in their 2012 paper ornithine was paired with lactose (Jain et al., 2012).

**Figure 8:**
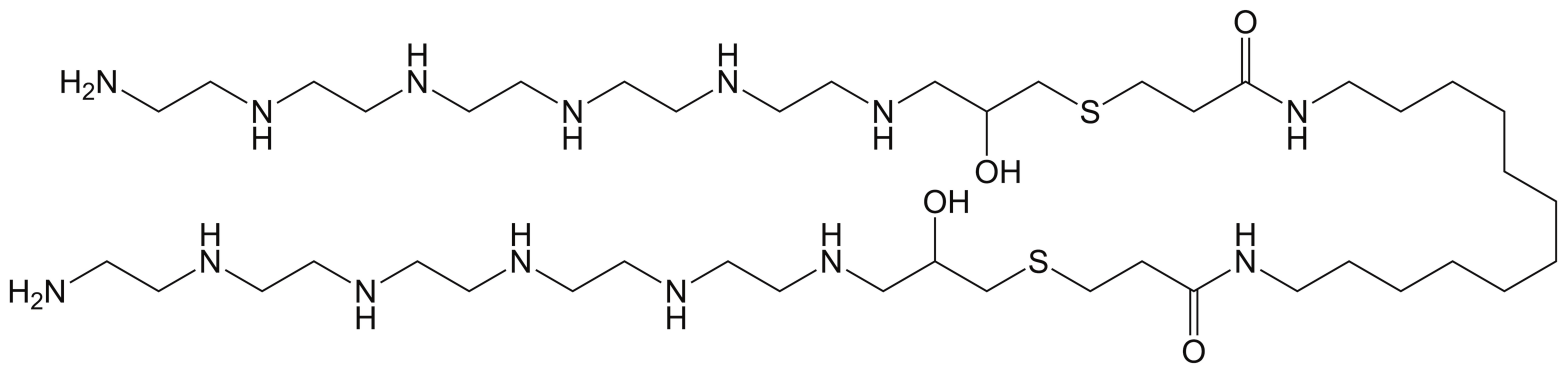
Symmetric BA featuring two hydrophilic pentaethylenehexamine head groups linked via a C12 hydrophobic tether by a thioether-based glycidyl group. Reproduced from Khan et al. (2012), Elsevier.

In 2012, Khan et al. published one of the first examples of a symmetrical cationic BA as a gene delivery agent (Khan et al., 2012). The full structure of their cationic BA is shown in Figure 8 and features a dodecane central hydrophobic moiety flanked by pentaethylenehexamine polar segments. The authors show that condensation of this BA with DNA at various N/P ratios results in bolaplexes with uniform dimensions (150–200 nm), competitive *in vitro* transfection relative to branched PEI, and marginal toxicity toward all cell lines tested.

Chen et al. (2013) took the concept of asymmetric BAs one step further by linking two distinct peptide head groups, RGD and R8, through a hydrophobic linker. The peptide-based BAs were shown to self-assemble into spherical nanoparticles in the presence of plasmid DNA. A comparative analysis revealed that the bola structure leads to more efficient DNA condensation vs. having the two peptide head groups linked directly together. In addition to efficient DNA condensation, the targeting allowed for enhanced gene delivery with both HeLa and 293T cell lines compared to PEI.

In 2015, a structurally simple BA was introduced by Paiva et al. (2015) that featured quaternary ammonium and primary alcohol head groups separated by a 22-carbon hydrocarbon linker. Here again, it was found that the BA can function as a standalone DNA binding material (bolaplex) or a component of a more complex formulation utilizing traditional cationic lipids such as 1,2-dioleoyl-3-trimethylammonium-propane (DOTAP). It was found that the BA/DOTAP coformulation stabilized the DNA complexes relative to DOTAP alone and did not adversely impact the transfection efficiency. Lastly, the BA/DOTAP lipoplexes exhibited lower toxicity vs. DOTAP alone.

**Figure 9:**
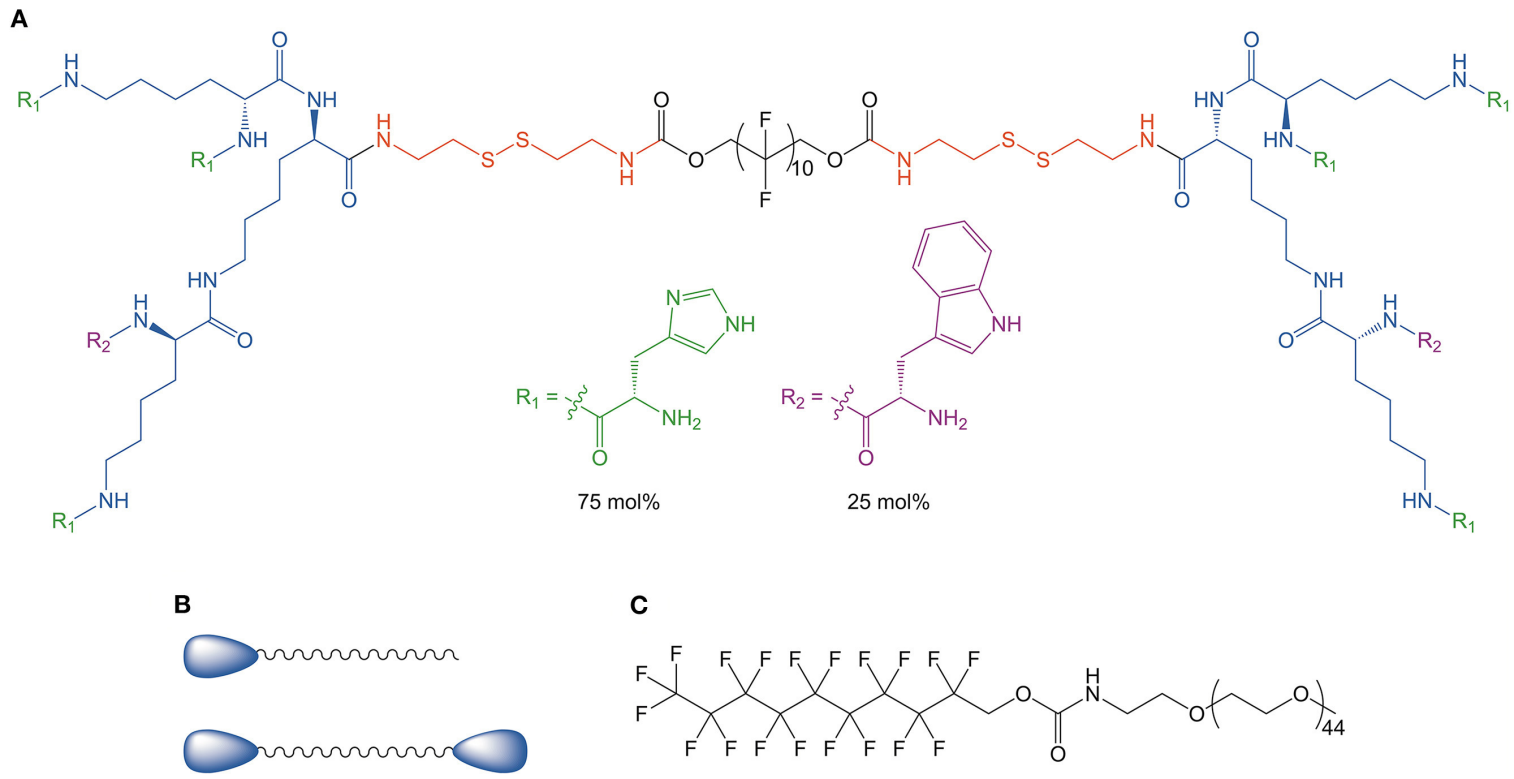
**(A)** Dendritic peptide structure utilizing a fluorocarbon central tether and branched hydrophilic head groups consisting of 75 mol% histidine and 25 mol% tryptophan. **(B)** Generic BA and monoamphiphile structure. **(C)** Structure of fPEG (2 K), the traditional monoamphiphile used for micelle coformulations. Reproduced from Eldredge et al. (2018), Elsevier.

The Guan group has adopted a highly systematic approach to designing BAs for siRNA delivery (see Figure 9) (Zeng et al., 2015; Eldredge et al., 2016, 2018). In order to optimize delivery as well as safety, four key design parameters were considered. First, the central hydrophobic linker was purposely chosen to be shorter than a native phospholipid bilayer such that the BA cannot directly insert into the cell membrane. This approach minimizes cytotoxicity. Second, the BA head groups were dendritic and consisted of 25 mol% tryptophan and 75 mol% histidine. The multivalency of the head groups promotes efficient complexation of the siRNA. The third design parameter involved the use of a fluorinated central linker to promote serum stability, cellular uptake, and good biodistribution. Lastly, a disulfide linker was used to connect the pH sensitive dendritic head groups with the hydrophobic segment. The reducing conditions of the cytosol facilitates disassembly of the BA-siRNA complexes and therefore allows for efficient release of the nucleic acids. By benchmarking these BA constructs against well-established vectors such as lipofectamine RNAiMAX, it was determined that the unique BA architecture was a key factor for complex stability, low cytotoxicity, and high transfection efficiency.

The Heldman group has been a leader in the development of BA-based targeted delivery systems featuring acetylcholine (ACh) head groups (Grinberg et al., 2008; Popov et al., 2010, 2012; Dakwar et al., 2012). In a series of joint papers between the Heldman and Shapiro groups (Kim et al., 2013, 2020; Gupta et al., 2015), the applicability of these ACh-containing BAs to siRNA delivery has been extensively explored. In 2013 (Kim et al., 2013), the first joint report described the *in silico, in vitro*, and *in vivo* delivery of siRNA. The BAs studied were based on the original design of Heldman: cationic acetylcholine head groups linked via a linear aliphatic chain. The initial study explored variation in linking geometry and correlated these small structural changes to siRNA complexation and transfection efficiency.

In 2015, the Heldman and Shapiro groups reported a new class of siRNA-delivering BA derived from jojoba oil–a liquid wax ester (Gupta et al., 2015). The structures of these amphiphilic materials are reminiscent of gemini surfactants due to the branching introduced during the installation of the cationic ACh groups. Having a lower density of cationic charge on the BA's terminal groups (one ACh group vs. two) led to enhanced transfection. In their most recent publication combining both simulation and experiment (Kim et al., 2020), the authors probed further the relationship between BA structure and siRNA delivery. Here combinations of structurally similar BAs gave the most desirable delivery properties. One BA bound the siRNA more tightly than the other thereby providing protection from nucleases yet diminishing transfection due to limited release of the nucleic acid cargo. If a second BA with weaker siRNA binding affinity was co-formulated, the release profile could be improved. The vesicles formed using the mixture of BAs complexed with siRNA, were shown to cross the blood brain barrier via transcytosis.

**Figure 10:**
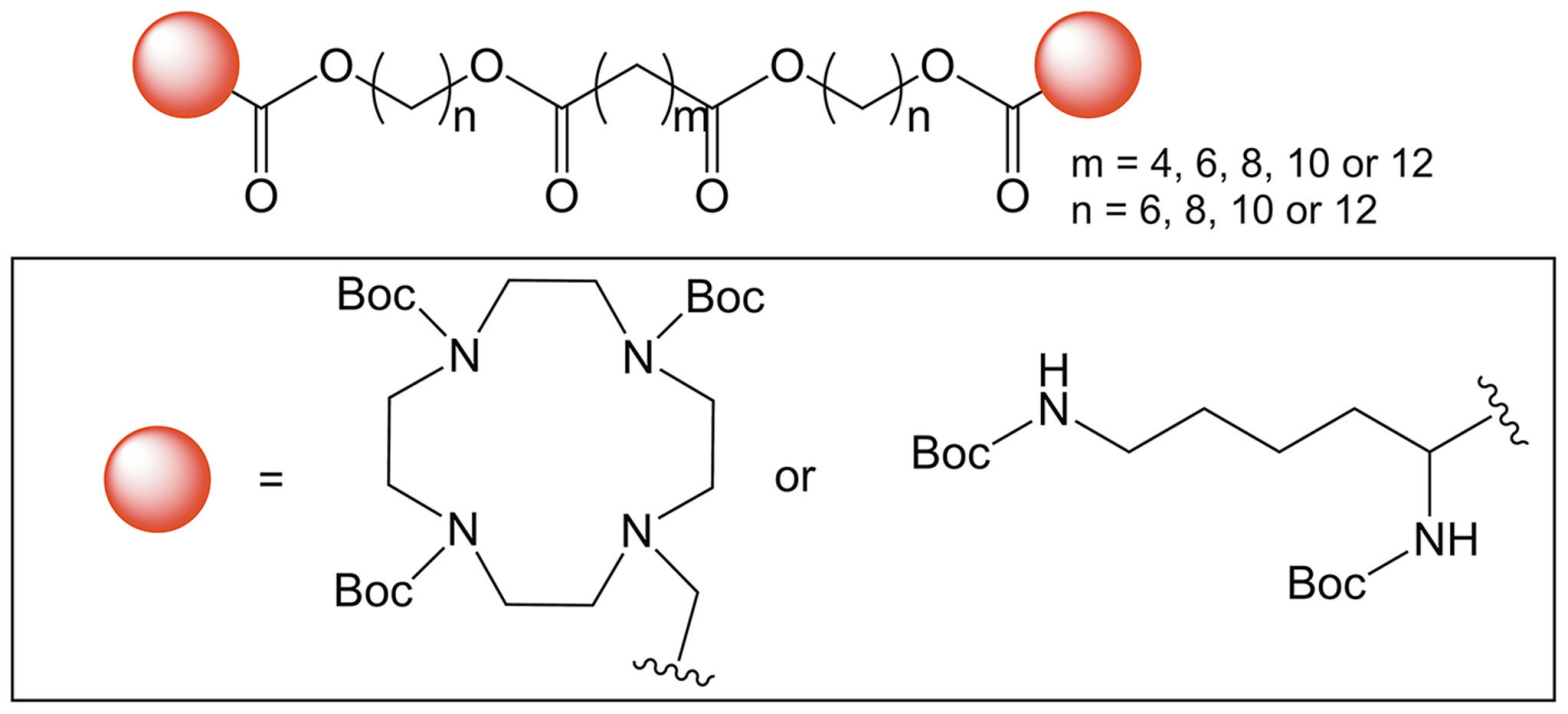
Synthetic BAs for bolasome coformulation with DOPE utilizing cyclen or lysine as hydrophilic head groups (Huang et al., 2016). Copyright 2016, Royal Society of Chemistry.

Huang et al. (2016) used the term “bolasome” to describe a mixed liposome containing both a BA and a more traditional helper fusogenic lipid–in this case 1,2-dioleoyl-sn-glycero-3-phosphoethanolamine (DOPE). The BAs employed were cationic and featured either lysine or cyclen polar head groups (see Figure 10). One finding in this paper was the possibility to balance high cationic charge with low cytotoxicity. Additionally, the authors found a correlation between hydrophobic chain length and successful liposome formation, DNA binding propensity, and transfection efficiency.

Unlike Huang et al., Gosangi et al. (2017) defined “bolasomes” as self-assembled structures made only from constituent BA and no helper lipid. In this same paper, the authors also studied “bolaliposomes,” BAs co-formulated with tocopheryl succinate-based cationic helper lipids. The co-formulated bolaliposomes outperformed the bolasome as well as the liposome containing only the tocopheryl succinate cationic lipids. MTT assays of the various combinations complexed with DNA revealed toxicity is sensitive to the cationic:anionic ratio in the formulation. Overall, however, the BA-containing bolaliposomes had relatively low toxicity when compared to lipofectamine 3K.

**Figure 11:**
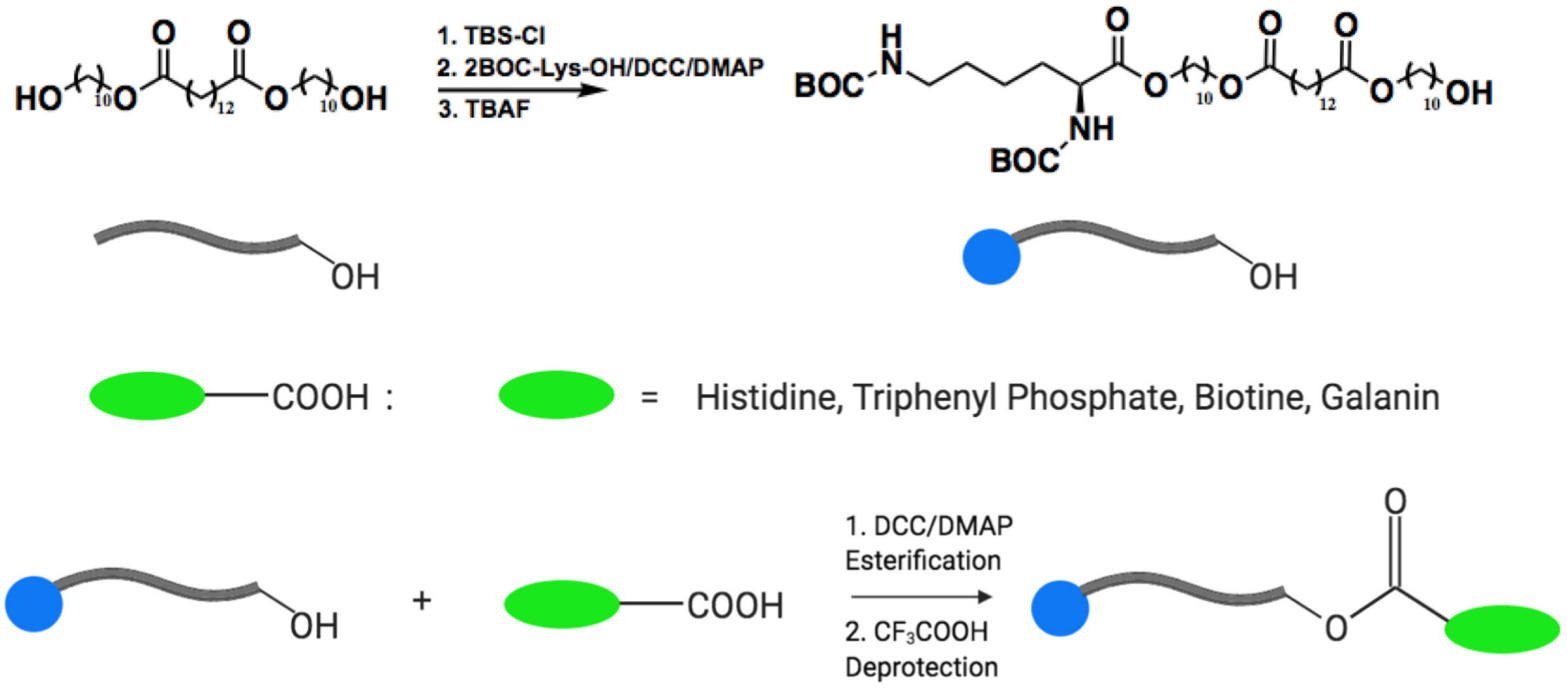
Huang et al. (2018) prepared a series of asymmetric BAs. One of the polar head groups was fixed as lysine while the second was chosen from an assortment of functional polar moieties. Copyright 2018, Multidisciplinary Digital Publishing Institute.

The majority of BAs are symmetric; however, there are scattered examples of asymmetric systems. In 2018, Yu et al. synthesized and studied the gene and drug delivery properties of both symmetric and asymmetric BAs (Huang et al., 2018). As is shown in Figure 11, lysine was used as a pH-sensitive cationic head group along with either another lysine (symmetric BA) or a functional moiety (asymmetric BA). Here, the BAs were co-formulated with DOPE both to increase the stability of the resulting self-assembled structures and to take advantage of the membrane fusion properties of DOPE and therefore increase cellular uptake. Both bolasomes and bolaplexes (a bolasome complexed with DNA) were synthesized and studied. One important conclusion was that the asymmetric BA-containing bolaplexes outperformed the symmetric versions in terms of transfection efficiency. Performance was also comparable to commercially available transfection systems. In particular, the BA-containing lysine and histidine polar head groups showed excellent uptake and endosomal release characteristics which were attributed to the high buffering capacity of the imidazole moiety.

**Figure 12:**
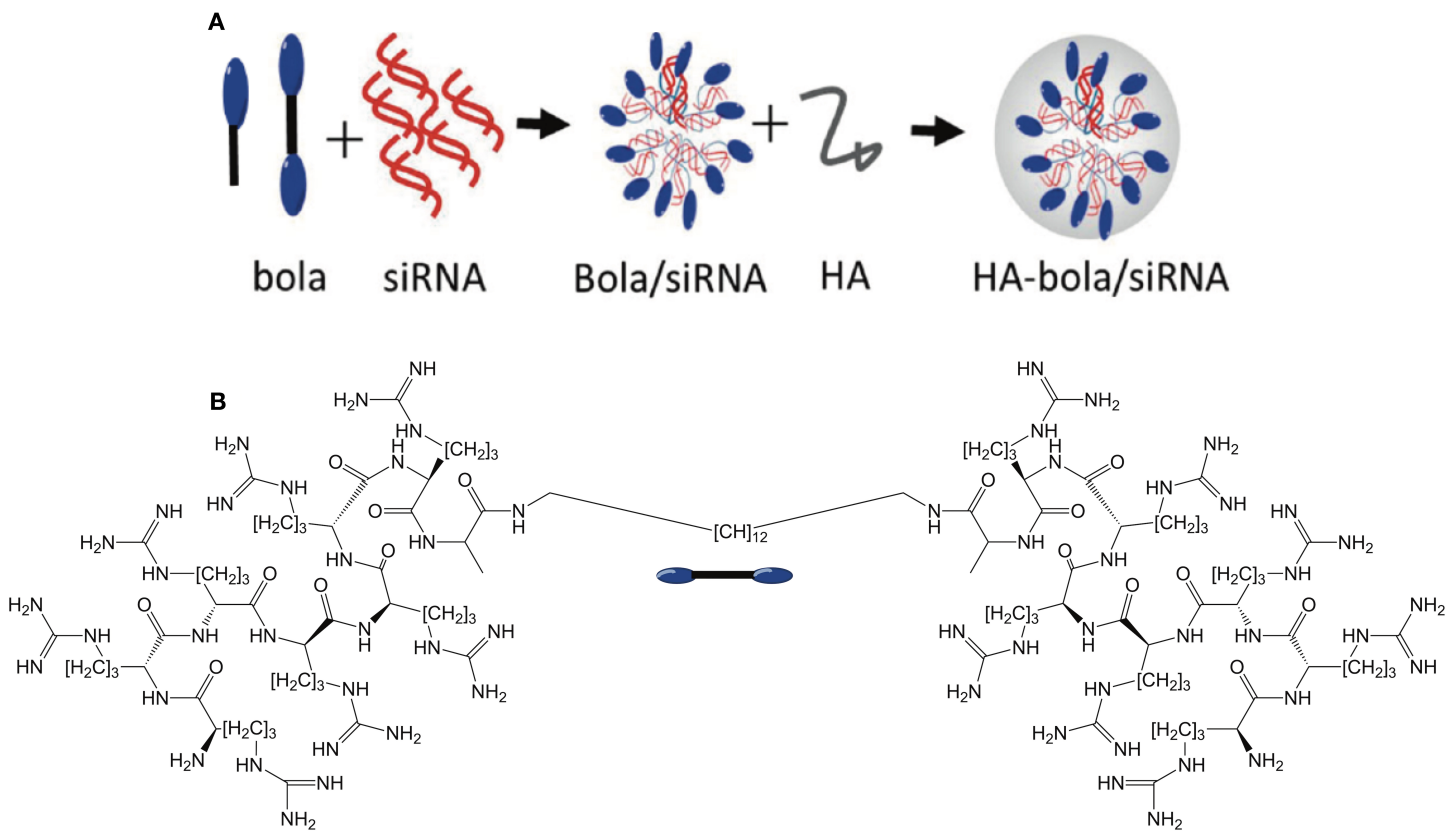
**(A)** Overview of bolasome formulation using the R8 BA or monoamphiphile to complex siRNA before coating with hyaluronic acid to confer a negative charge. **(B)** Structure of the R8 bolaamphiphile. Reproduced from Jia et al. (2020), American Chemical Society.

In a very interesting approach to siRNA delivery, Jia et al. (2020) synthesized a BA with cell penetrating peptide octa-arginine (R8) head groups (see Figure 12). A control monoamphiphile was also prepared in order to probe the impact of the bola structure on delivery efficiency. The R8 BAs complexed siRNA more efficiently than both the R8-mono amphiphile and PEI. In addition, the R8-BA showed higher biocompatibility than the R8-monoamphiphile. In order to further increase the functionality of these gene delivery agents, the cationic BA/siRNA complexes were overcoated with negatively charged hyaluronic acid (HA). The purpose of the HA overcoat was two-fold. First, HA is known to impart “stealth” characteristics to the particles by shielding the positive surface charges and avoiding non-specific uptake by the reticuloendothelial system thus increasing the *in vivo* stability. Second, HA targets the CD44 receptors overexpressed on the surface of cancer cells. The authors suggest that the HA coating is degraded by hyaluronidase once reaching the target tumor tissue thereby revealing the R8 cell penetrating feature. Overall, this is a versatile system that demonstrates the full potential of BAs as targeted gene delivery agents.

**Figure 13:**
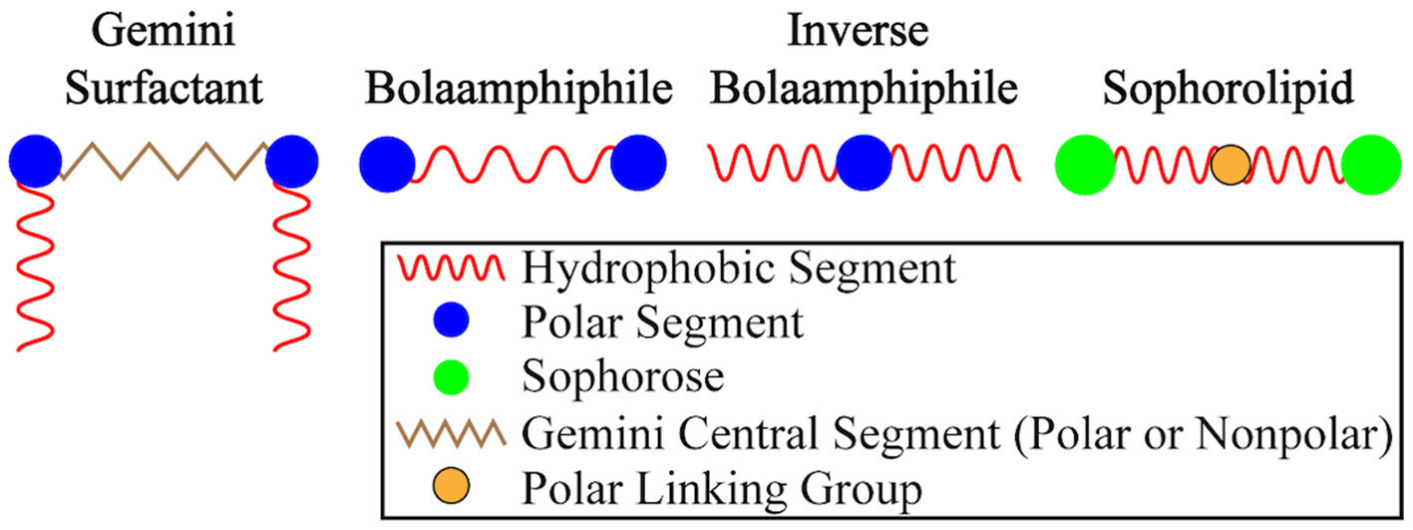
A graphical summary of BA variants. Traditional BAs have hydrophobic central units while inverse BAs feature polar/hydrophilic central segments.

The segmented architecture of a traditional A-B-A-type BAs feature hydrophilic capping moieties linked through a hydrophobic tether. Inverse BAs, on the other hand, feature a hydrophilic central segment flanked by hydrophobic groups (see Figure 13). Like traditional BAs, inverse BAs can be synthesized with a symmetric or asymmetric architecture. Gemini-type lipids (Grenier et al., 2012), including gemini BAs, feature a traditional hydrophobic central linker but add additional flanking hydrophobic segments linked through cationic groups.

**Figure 14:**
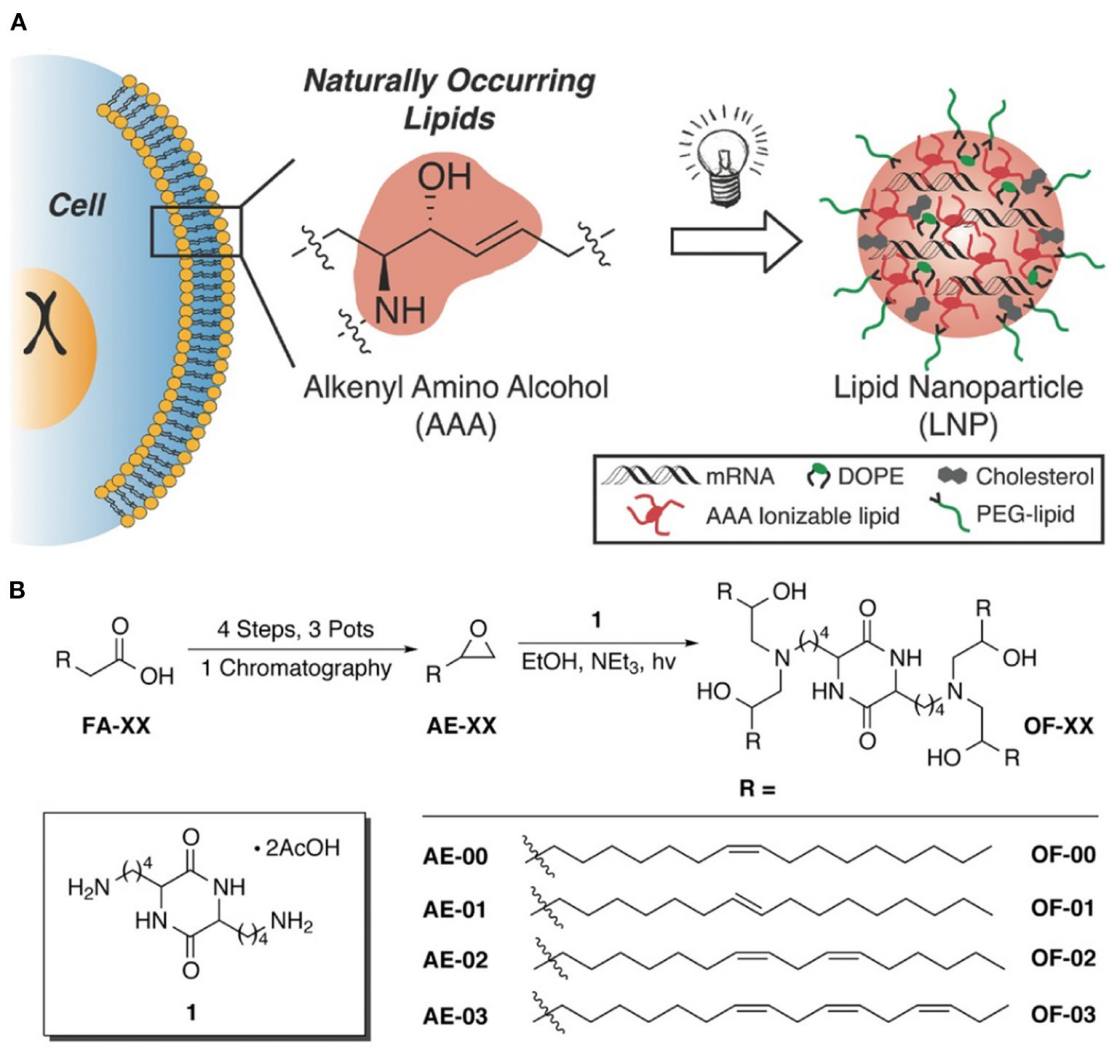
**(A)** Discovery of alkenyl amino alcohols (AAA) in cell membranes serve as the basis for synthesis of inverse BAs for mRNA delivery. **(B)** 5-step synthesis of inverse BA containing hydrophilic AAA moieties flanked by various fatty acid tails (Fenton et al., 2016). Copyright 2016, Advanced Materials.

Like traditional BAs, inverse BAs have been shown to complex nucleic acids. For example, in 2016 Anderson and Langer reported a class of alkenyl amino alcohol ionizable lipids for mRNA delivery (see Figure 14) (Fenton et al., 2016). These structures feature a polar central unit with an ionizable amino alcohol moiety flanked by branched hydrophobic segments containing unsaturated fatty acids. These materials, when co-formulated with other more traditional lipids such as cholesterol and DOPE, were able to outperform benchmark compounds.

The Siegwart group explored zwitterionic amino lipids (ZALs) to delivery long RNAs (Miller et al., 2017). These structures are highly branched yet the non-polar and polar segments are somewhat segmented. Miller et al. evaluated the potential of these materials to deliver sgRNA for Cas9-based *in vivo* gene editing via surveyor nuclease assay and a fluorescence assay, respectively.

The Whitehead group made further progress in RNA delivery using branched tail lipid nanoparticles having an inverse BA-type architecture (Hajj et al., 2020). This particular type of lipid was shown to co-deliver, *in vivo*, three unique mRNAs. The delivery system was potent, non-toxic, and non-immunogenic. Additionally, the inverse BA nanoparticles transfect all liver cell lines with 86–88% efficiency. Furthermore, inverse BA nanoparticles maintained high efficacy through repeat dosing without inducing an immune response.

Inspired by the structure of BA-based nanoparticles, Martinez-Negro et al. (2018) evaluated the potential of gemini-BAs for *in vitro* pDNA delivery. In contrast to traditional monovalent surfactants, geminis have a much lower critical aggregate concentration allowing for the formation of smaller nanoparticles while using less material (Damen et al., 2018). Additionally, the presence of two head groups opens the possibility for multivalent interactions with a substrate or biological target. The gemini BAs were formulated with DOPE. Gel electrophoresis shows strong pDNA binding and protection. Transfection efficiencies were similar to Lipo2000^*^ when co-formulated with DOPE.

## Antimicrobial Approaches Involving Bolaamphiphiles

Antimicrobial resistance (AMR) is a threat to global health (Bush et al., 2011; Baral and Mozafari, 2020). One approach to combat this issue is the use of membrane active antimicrobials. In contrast to traditional antibiotics that interfere with bacterial biosynthesis, membrane active antimicrobials target the conserved structure of the bacterial membrane (Li et al., 2015). The bacterial membrane is considered “evolutionarily difficult” to respond or adapt to disruptions and thereby minimizes resistance against agents that target it. The segmented domain structure of BAs are, more often than not, membrane active and can lead to potent antimicrobial activity. The cationic outer segments of a BA along with its hydrophobic tether allows for complementary binding opportunities to bacterial membranes.

Sauvage et al. (2019) studied DQ–a well-known permanently cationic BA. DQ is symmetrical, with a hydrophobic alkyl chain core and two charged end groups. DQ was synthesized in the 1950s, but more recently, it has been investigated for its mitochondrial targeting properties. Sauvage et al. discovered that DQ causes electrostatic repulsion between lipid bilayers leading to membrane swelling. Additionally, the U-shape structure DQ adopts when inserted into the bilayer causes fragmentation of liposomes into smaller vesicles. The authors findings related to the stability of DQ-membrane interactions, especially under physiological conditions, should prompt more research into the appropriate formulation of DQ for mitochondrial delivery applications.

**Figure 15:**
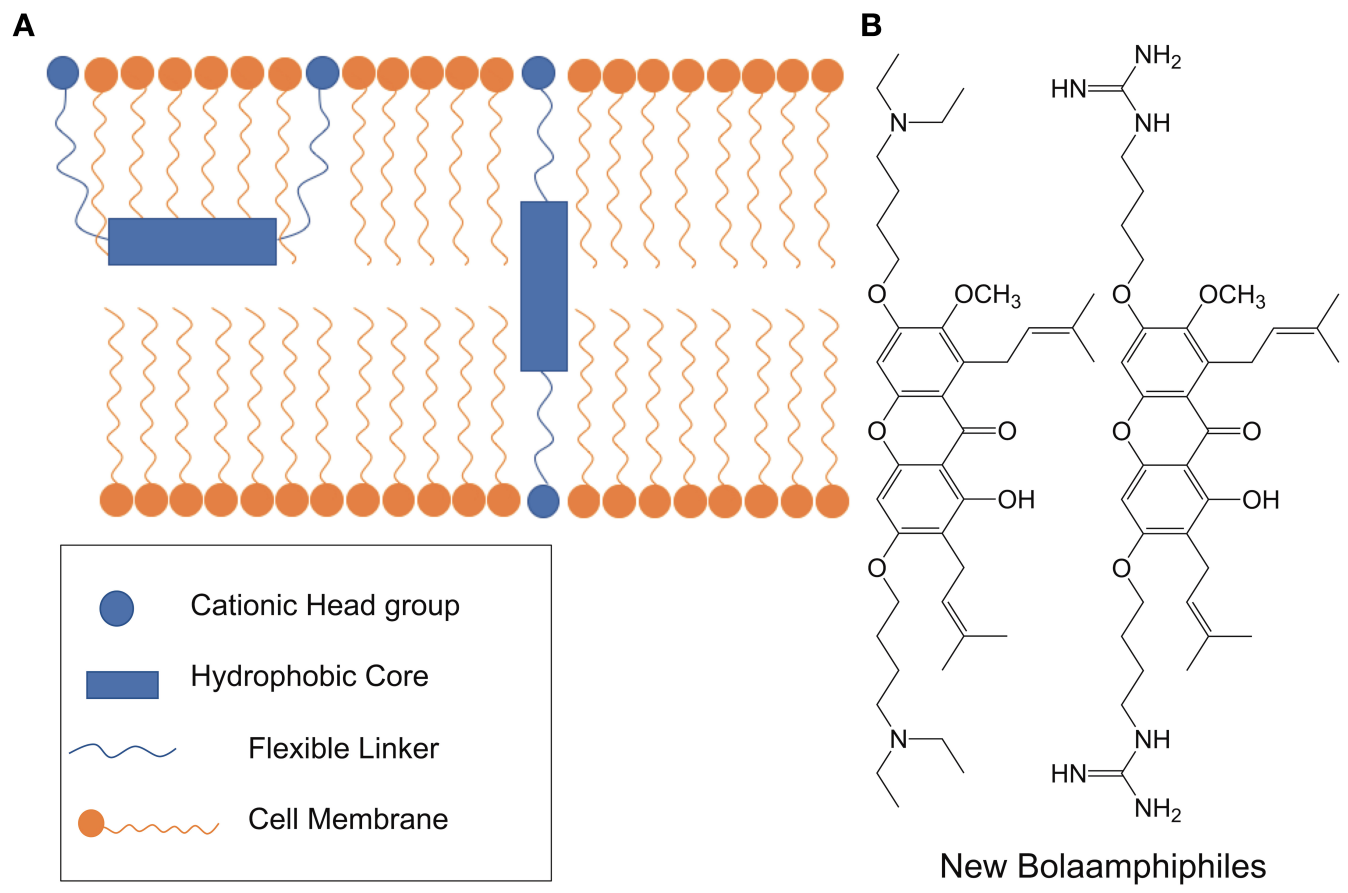
**(A)** Using molecular dynamics and biophysical methods, Li et al., proposed a three-step model for the interaction of BA-like antimicrobials with gram positive pathogens. Initially the xanthone-based BA is adsorbed in a U-shaped conformation but eventually adopts a highly disruptive transmembrane configuration. **(B)** Structures of xanthone-based BA antimicrobials. Reproduced from Li et al. (2015), Elsevier.

In 2015 the groups of Verma and Beuerman put forth a generalizable BA pharmacophore model for membrane active antimicrobials (see Figure 15) (Li et al., 2015). The authors combined molecular dynamics with biophysical and microbiological studies in order to arrive at a three-step mechanistic model for BA-type molecules: adsorption, translocation, and disruption. The study focused on alpha-mangostin hydrophobic core species but the generalized design principles apply to other BA-type antimicrobials.

In 2016 the Gokel group studied a collection of *bis*(tryptophan)s including several BA-type structures with central hydrophobic tethers ranging from 3 to 12 carbons (Meisel et al., 2016). The BA with the longest hydrophobic linker, 12 carbons, had the greatest antimicrobial activity out of all the *bis*(tryptophan)s but it was also cytotoxic.

Transcription factor decoys (TFD) block essential microbial transcription and therefore represent a powerful weapon against antimicrobial resistance (Marin-Menendez et al., 2017). BAs have emerged as an important component of oligonucleotide delivery systems (not the active antimicrobial). In two separate papers, Mamusa et al. demonstrate that cationic BA [12-*bis*-THA]Cl_2_, which is structurally similar to dequalinium (Weissig et al., 1998), forms nanocomplexes with model TFD (Mamusa et al., 2016, 2017). The authors formulated 12-*bis*-THA in a liposomal carrier and showed that helper fusogenic lipids are required to promote bacterial cell entry and transfection. Although the BA performed its delivery function, it was also correlated with non-negligible cytotoxicity.

Gonzalez-Paredes et al. (2019) took a slightly different approach to TFD delivery while still retaining 12-*bis*-THA as the oligonucleotide binding agent. Preformed anionic solid lipid nanoparticles were synthesized and overcoated, sequentially, with 12-*bis*-THA (cationic) then the particular TFD. It was found that nanocomplexes formed in this way preferentially accumulated in bacteria.

In 2016, Hegarty et al. reported the first *in vitro* antisense treatment to inhibit *Clostridium difficile* using a bolasome-based approach (Hegarty et al., 2016). Cationic dequalinium derivatives were employed and their ability to form antisense nanocomplexes was studied. In a follow-up 2018 paper, that once again utilized cationic BAs as oligonucleotide-binding agents, Sharma et al. (2018) showed that cationic BA vesicle-like aggregates are promising nanocarriers for *C. difficile* therapy. The authors identify several areas still needing more research including: structure-property relationships in these delivery systems, toxicity, and *in vivo* testing.

**Figure 16:**
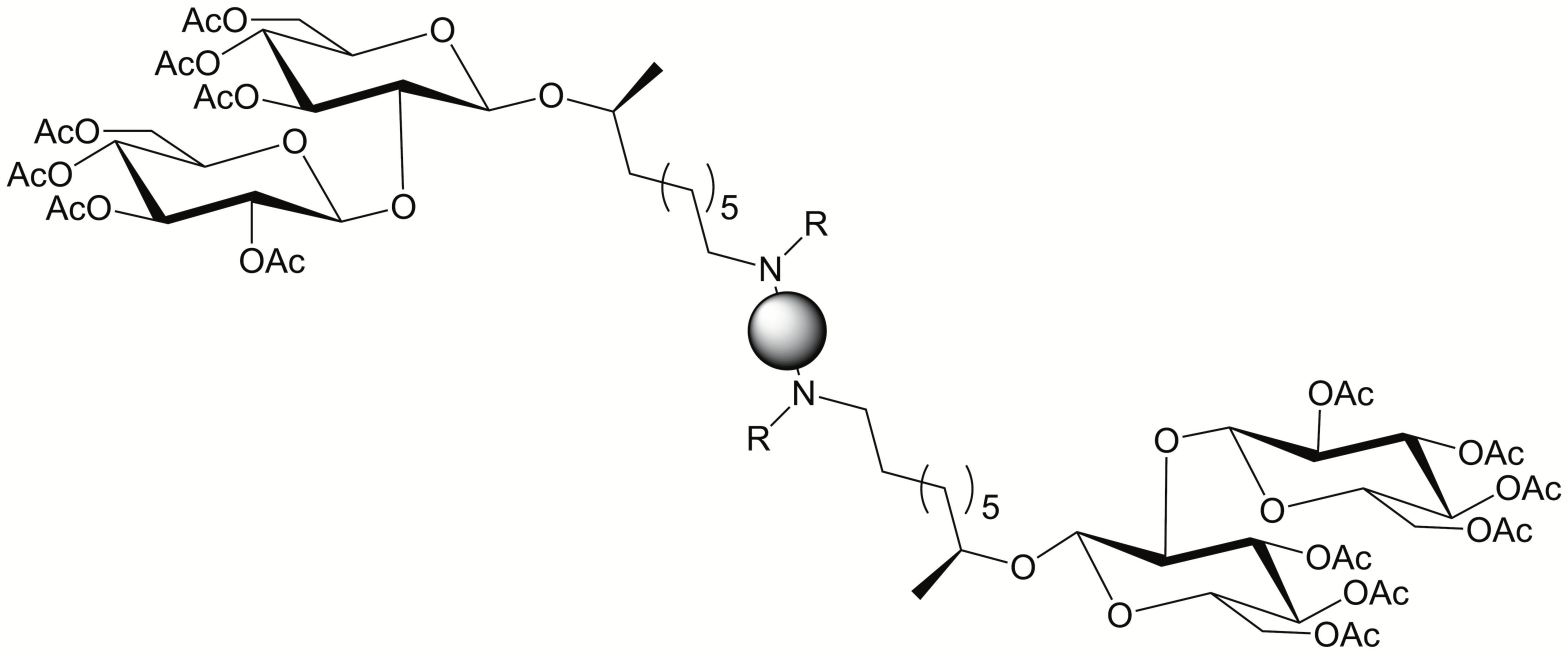
Sophorolipid BA general structure. Reproduced from Delbeke et al. (2018), American Chemical Society.

An emerging class of BAs are those derived from sophorolipids (Cuvier et al., 2015; Baccile et al., 2017). Sophorolipids are a renewable class of glycolipid biosurfactant derived from *Starmerella bombicola* yeast. Delbeke et al. (2018) studied sophorolipid BAs for their antimicrobial activity and their potential as transfection agents (see Figure 16). The majority of sophorolipid BAs studied showed activity against gram positive bacteria. DNA binding, when co-formulated with DOPE, was weak and transfection was inefficient. Although transfection was poor, cytotoxicity was negligible.

## Bolaamphiphilic Prodrugs

Prodrugs are derived from their pharmacologically active precursors but are chemically altered to achieve efficient, and sometimes sustained, delivery (Testa, 2004; Rautio et al., 2008). The prodrug, or inactive form, more easily finds its biological target or escapes degradation only to reveal the active form of the molecule through a triggering event. In the case of intracellular delivery, prodrugs must have low cytotoxicity and gain access to the cytosol through endocytosis or various receptor-mediated mechanisms. BAs are especially qualified for this type of application as their rich self-assembly characteristics (Meister and Blume, 2007; Dhasaiyan and Prasad, 2017) and membrane active properties (Gokel and Murillo, 1996; Gill et al., 2020) facilitate cellular uptake.

**Figure 17:**
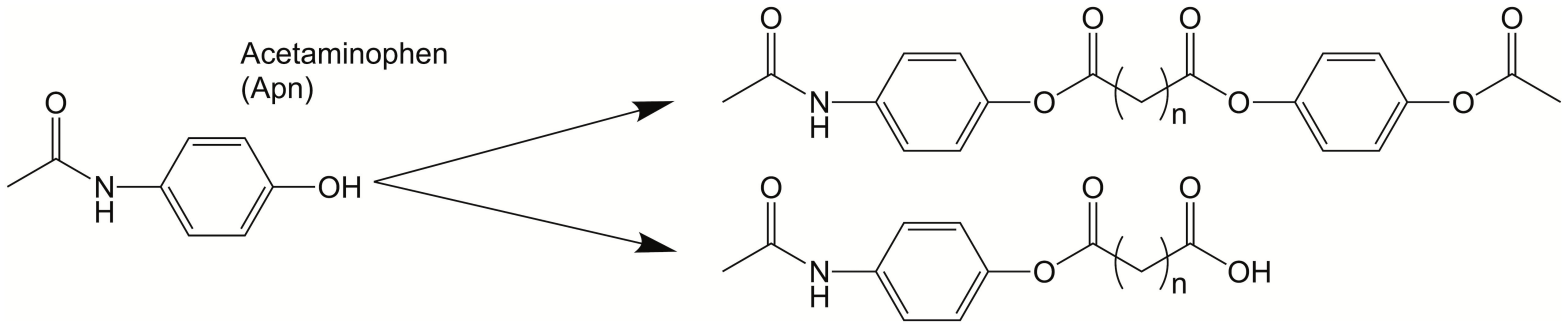
Acetaminophen was modified into a bola-like structure. These amphiphilic derivatives self-assemble into hydrogels that can act as drug delivery platforms. Reproduced from Vemula et al. (2009), Elsevier.

The groups of Karp and John synthesized a series acetaminophen prodrugs featuring hydrolysable and non-hydrolysable BAs (see Figure 17) (Vemula et al., 2009). The self-assembly of these acetaminophen prodrugs promotes hydrogelation. Fortunately, enzymatic action on the hydrogel results in liberation of the active form of acetaminophen. The authors showed modulation of the release kinetics based on both the temperature and enzyme concentration. Lastly, the authors reveal the ability to co-encapsulate a second bioactive molecule in the self-assembled hydrogels. In studies involving the treatment of mesenchymal stem cells with this particular self-assembled hydrogel, the acetaminophen prodrug hydrogels did not interfere with cell viability, adhesion, or proliferation.

Unlike the example by Karp and John where the BA utilizes one prodrug, others have synthesized asymmetric BAs in order to achieve combination therapy. Jin et al. (2010, 2011) proposed a new approach to improve the low bioavailability and weak targeting in some antivirals. A BA prodrug was synthesized featuring two anti-HIV drugs as head groups–zidovudine and didanosine. The anti-HIV drugs were linked by a deoxycholyl spacer. The resulting asymmetric BA was reported to be soluble in standard organic solvents and self-assemble into spherical vesicles that exhibited negative zeta potentials. The BA-based nanoassemblies were soft monolayers which bend to form closed vesicles. This work shows that two different drugs can be co-delivered to their cellular target via a single BA molecular construct.

**Figure 18:**
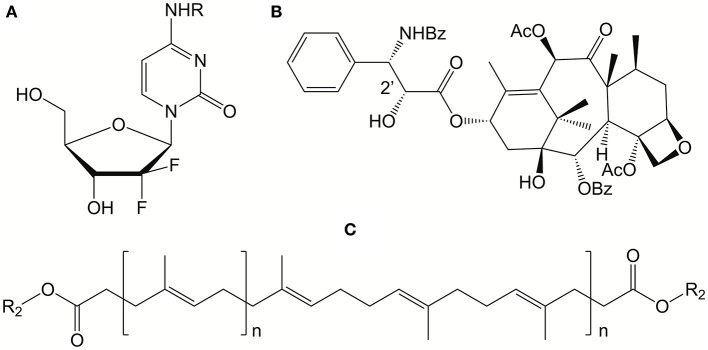
Structures of antitumor drugs, gemcitabine **(A)** and paclitaxel **(B)**, where R binds to the proposed hydrophobic squalene backbone **(C)**. These “bolaform” prodrugs were shown to form nanoassemblies with *in vitro* activity on human and murine cancel cell lines. Reproduced from Caron et al. (2014), Royal Society of Chemistry.

In a similar approach, Caron et al. synthesized asymmetric BAs composed of Paclitaxel and gemcitabine (both antitumor agents) that were joined by a hydrophobic isoprene linker (see Figure 18) (Caron et al., 2014). These BA co-drug structures were superior to free drugs, combinations of each prodrug, and single-drug structures, forming nanoassemblies with high payloads and improved *in vitro* activities.

## Hydrogels Containing or Derived from Bolaamphiphiles

Hydrogels offer a unique pathway for the conversion of small molecules (low molecular weight gelators) into macromolecular structures. BAs are attractive building blocks for hydrogels as their segmented amphiphilicity promotes both hydrophobic and polar interactions (e.g., hydrogen bonding) leading to a rich design space. The self-assembly proclivities of these materials are diverse and influenced by a portfolio of weak interactions including hydrogen bonding, π-π interactions, aromatic interactions, and hydrophobic effects.

Romera et al. (2018) show how BA self-assembly mimics the cytoskeletal network by allowing for both strain-stiffening and self-healing characteristics. The BAs utilized in this study undergo a hierarchal self-assembly process where the hydrophobic domain (aliphatic linkers joined centrally through a butadiyne moiety) stack and subsequently form strands. Incorporating a fraction of chemically addressable end groups into these self-assembled ribbons allows for chemical crosslinking via copper-catalyzed azide-alkyne chemistry. Unlike polyisocyanopeptide strain-stiffening hydrogels (Jaspers et al., 2016), the BA-type hydrogels are covalently crosslinked (as opposed to aggregated) which not only avoids clumping but also permits tuning of the pore size.

Photodynamic therapy is another potential solution to the onset of multi-drug resistant bacteria (Celli et al., 2010; Chen et al., 2020). This therapeutic modality uses photosensitizers, light, and oxygen to disable the resistance-building capability of bacteria. In 2019, Goergen et al. used single chain BAs as a gelating agent (Goergen et al., 2019) The BAs used by Goergen feature a 32-carbon central hydrophobic domain linked by either two phosphocholine or two dimethylphosphoethanolamine head groups. The BAs were co-formulated with methylene blue–a photosensitizer. The stability of the self-assembled hydrogels decreased with minimal shear forces. Aerogels, derived from the self-assembled BA-containing hydrogels, were fabricated and provide convenient access to a shelf-stable form of the therapeutic material.

A growing area of interest relates to nucleotide BAs. Seminal work by Shimizu et al. showed spontaneous fiber formation using 3‘-phosphorylated thymidine moieties connected to both ends of a long oligomethylene spacer (Iwaura et al., 2002). More recently, the Barthélémy group has pioneered glycosyl-nucleoside BAs (GNBA) (Latxague et al., 2015, 2016; Ramin et al., 2017; Baillet et al., 2018, 2020; Bansode et al., 2020). For example, in 2016, Latxague et al. studied carbamate-based GNBA gelators (Latxague et al., 2016). The thymidine-based hydrophilic head groups flank a 12-carbon alkyl chain linked via carbamate functional groups. Hydrogels could be formed with 4% solutions (w/v) and were stable at room temperature. The self-assembled gels exhibited an elastic character (G' > 50 kPa) greater than similarly reported ether analogs (G' ≈ 30 kPa) due to the extensive hydrogen-bonding network. The gels exhibit dynamic properties such as reversible liquification under stress. The materials thixotropic properties and gel-sol characteristics open-up opportunities in injectable drug delivery applications. Barthélémy et al. have also reported GNBAs with urea linkages (Ramin et al., 2017). Overall, Barthélémy has suggested that BA-based low molecular weight gelators offer certain mechanical and biological advantages over more commonly used natural polymers such as alginate or hyaluronic acid. Those advantages include: control over structure and purity, easy handling prior to gelation, and excellent mechanical stability including limited shrinkage due to syneresis.

Returning to the theme of light-responsive BA-containing hydrogels, Baillet et al. (2020) developed light sensitive low molecular weight gelators from GNBAs. The central hydrophobic unit embedded a photosensitive stilbene moiety that, when photoswitched, initiated a gel-sol transition. The gels made from these light-responsive BAs feature high elastic moduli and thixotropic behavior thereby opening-up possible applications as drug delivery systems.

**Figure 19:**
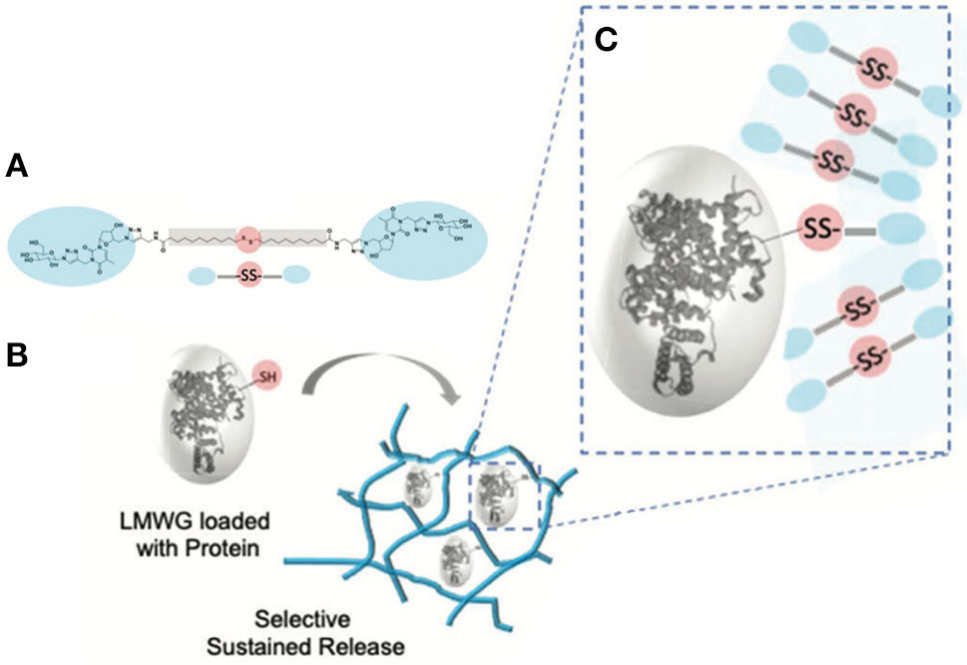
**(A)** Glycosylated nucleoside based bolaamphiphiles (GNBA) with a disulfide function (SS-GNBA-3) resulting in supramolecular assembly of the SS-GNBA-3 via weak interactions, stabilizes a low molecular weight gel (LMWG). **(B)** The LMWGs loaded with proteins obtained in the presence of biomolecules. **(C)** Selective sustained release from gel, protein's covalent attachment via disulfide bridges between protein and supramolecular network (Bansode et al., 2020). Copyright 2020, Royal Society of Chemistry.

Bansode et al. (2020) developed a redox responsive GNBA (Figure 19). Here, the supramolecular interactions of the amphiphilic GNBA stabilize droplets and fiber-like networks. Disulfide bonds present in the network can be cleaved by reducing agents (e.g., DTT) to promote disassembly or protein interchange. The disulfide interchange between the matrix and a protein was used to anchor the protein to the gel network and thereby slow its release.

A new class of symmetrical sophoroside BA was recently introduced (Van Renterghem et al., 2018) and studied (Baccile et al., 2018). This glyco-BA features two sophorose head groups on each end separated via a C16:0 spacer. Baccile et al. (2018) studied the self-assembled fibrillar network produced by these fascinating BAs including their temperature dependent behaviors. The self-assembly of the BA forms structures with a 20 nm diameter and 100 nm length. These authors were the first to analyze the elastic module of any bio-based glycol-amphiphile and found that temperature control can adjust the elastic modulus from 100 Pa to 20 kPa. The biobased route allows for large scale production and entry into the consumer and biomedical technologies areas.

The Yan group studied drug-loaded hydrogels derived from rationally designed bola-dipeptides (Zou et al., 2020). Like many of the BA-based hydrogels discussed in this section, these peptide-containing hydrogels exhibit good flow under shear stress, excellent recovery properties, and sustained release profiles. In this particular case, hydrogels were loaded with the prodrug 5-aminolevulinic acid hydrochloride that, upon release from the hydrogel, biosynthetically initiate the production of an active photodynamic therapeutic agent.

**Figure 20:**
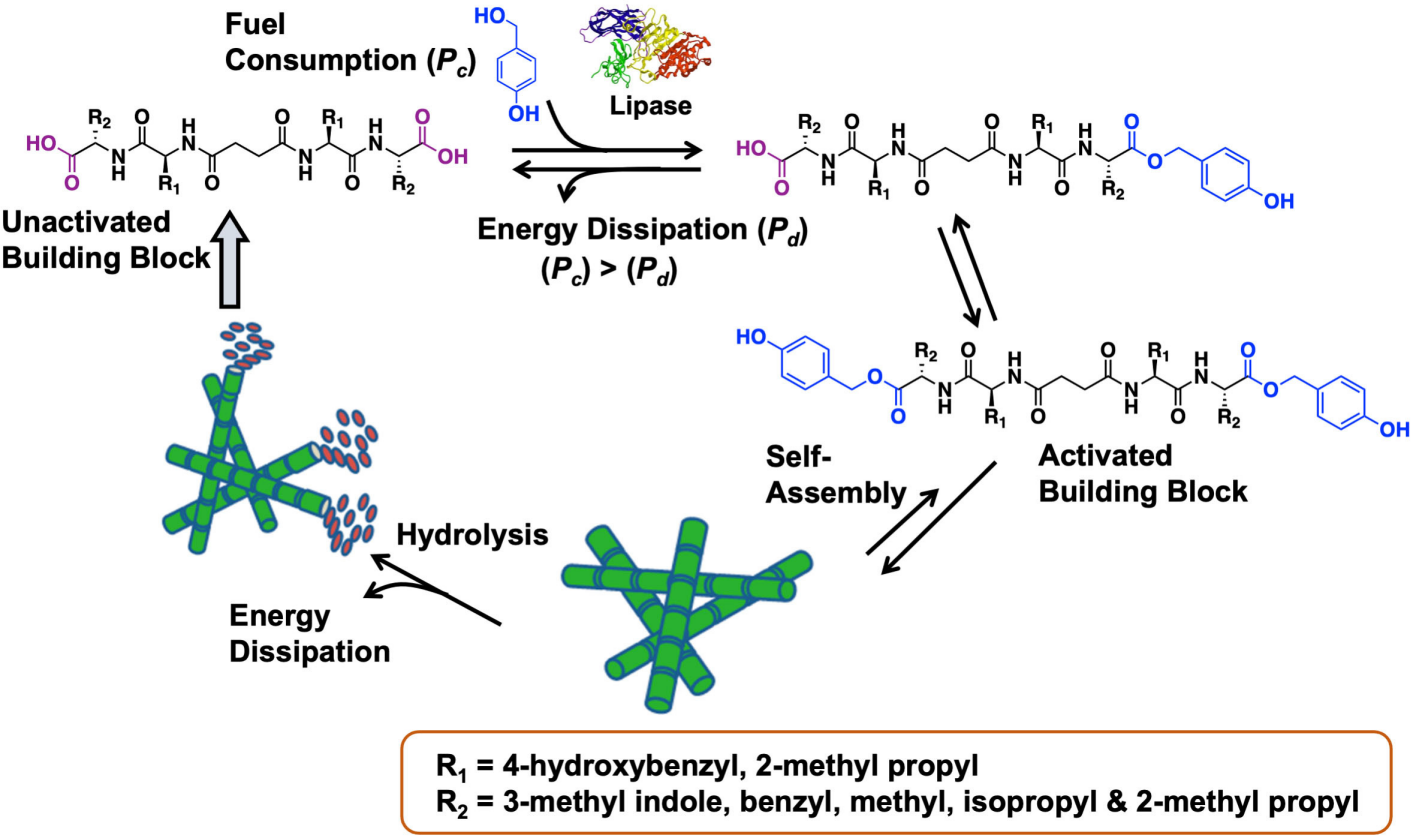
Enzymatic activation of a peptide-based BA leads to the self-assembly. After fuel consumption, peptide diesters form which then self-assemble into fibrillar network leading to stable hydrogels. The hydrogels collapse back into the inactivated parent BA dissipating the fuel through hydrolysis. The hydrogel exhibited favorable properties to support and promote the growth of human umbilical cord stem cells. Figure adapted from Das et al. (2015).

Das et al. have pioneered work in peptide-based BAs (Maity et al., 2012, 2014; Das and Gavel, 2020). In a 2015 study, dissipative peptide BAs were reported in which enzyme-activated amphiphiles assemble into robust hydrogels (see Figure 20, Das et al., 2015). The peptide BAs were synthesized in good yields through traditional solution phase coupling methods. In this out-of-equilibrium peptide-based system, carboxylic acid end groups were esterified with benzyl alcohol in the presence of lipase. Hydrogen bonding and pi stacking interactions facilitate the self-assembly of these peptide precursors into nanofibrillar hydrogels. The fiber dimensions were ~16 nanometers in width by several micrometers in length. Energy dissipation is achieved through hydrolysis of the ester end groups and reversion back to the original BA. In terms of biomaterials relevance, the self-assembled BA-based hydrogels were found to promote human umbilical cord stem cell proliferation as well as survival. Overall, this is a nice demonstration of how triggered self-assembly (and disassembly) of BAs can be achieved using enzymatic approaches.

## Conclusions

The unique segmented amphiphilicity and membrane active properties of BAs creates a rich design space for new therapeutics and delivery systems. The structural diversity of BAs is expanding well beyond the traditional linear A-B-A motifs. Dendritic or polymeric head groups, for example, are increasingly common as they promote multivalency–an important design consideration for engaging cell and/or protein interactions. Significant advances have also been made in the use of BAs as gene delivery agents, however, important questions remain. Is it more effective (and safe) to use BAs as a standalone nucleic acid complexing agent (bolaplex) or as just one ingredient of a multi-component lipid formulation? BA-based hydrogels also remain an exciting area of research where creative design can produce multifunctional materials with dynamic properties. Overall, innovative synthetic design will continue to drive discovery. The synthesis of new BA constructs with intermediate molecular weights, falling somewhere between traditional small molecule BAs and segmented polymers, is interesting and somewhat underdeveloped.

## Author Contributions

JH, AM, CW, GV, and PI wrote the paper. All authors contributed to the article and approved the submitted version.

## Conflict of Interest

The authors declare that the research was conducted in the absence of any commercial or financial relationships that could be construed as a potential conflict of interest.
